# The Provision of Genetic Testing and Related Services in Quebec, Canada

**DOI:** 10.3389/fgene.2020.00127

**Published:** 2020-03-04

**Authors:** Brigid Unim, Corrado De Vito, Julie Hagan, Paolo Villari, Bartha Maria Knoppers, Ma’n Zawati

**Affiliations:** ^1^ Department of Public Health and Infectious Diseases, Sapienza University of Rome, Rome, Italy; ^2^ Centre of Genomics and Policy, McGill University, Montreal, QC, Canada

**Keywords:** delivery model, genetic service, genetic testing, care pathways, policy, evaluation

## Abstract

**Background:**

Research in the field of genomics and genetics has evolved in recent years and so has the demand of consumers who are increasingly interested in genomic prediction of diseases and various traits. The aim of this study is to identify genetic service delivery models, policies governing the use of genomics medicine, and measures to evaluate genetic services in the province of Quebec, Canada.

**Methods:**

An *ad hoc* questionnaire was designed and administered online in 2017 to healthcare workers with good knowledge or experience in the provision of BReast CAncer genes 1 and 2 (BRCA1/2), Lynch syndrome, familial hypercholesterolemia, inherited thrombophilia genetic tests, engaged in policy planning or evaluation of genetic services. A quali-quantitative analysis of the survey results was performed.

**Results:**

Thirty professionals participated in the study. The delivery models are classified in five categories according to the leading role of healthcare professionals in patient care pathways: i) the geneticist model; ii) the primary care model; iii) the medical specialist model; iv) the population screening program model; and v) the direct-to-consumer model. Barriers to genetic services are the coverage of genetic tests by the publicly funded healthcare system, the availability of qualified personnel, and the number of genetic centers. Regulatory oversight concerning the provision of genetic services appears to be insufficient.

**Conclusions:**

Integration between genetics and the overall healthcare system in Quebec is in an early phase. Current models of genetic services require good level of genetic knowledge by all medical specialists, collaboration among different healthcare personnel, and work redistribution. The proper implementation of genomics into healthcare can be achieved through education and training, proper regulatory oversight, genomic policies, and public awareness.

## Introduction

Personalized medicine has been adopted worldwide after the completion of the first sequence of human genome in 2003 (Collins et al., 2003). This had led to an increase in the development of genetic tests used in routine clinical practice and research activities. Despite the fast and promising development of genomic applications, there are concerns about how to ensure high standards of genetic services (Cassiman, 2005; Khoury et al., 2007; Scheuner et al., 2008; Liehr et al., 2017; Skirton, 2017). Of particular concern is the lack of quality criteria of genetic service delivery models, which are components of the Public Health Genomics (PHG) framework. A genetic service delivery model combines healthcare services for individuals (i.e., diagnosis and treatment of genetic disorders) and public health services and activities (i.e., population-based screening, policy making, financing, information and education of healthcare workers and the general population, service performance assessment, and research) (Unim et al., 2017). Another concern is the early introduction in practice of applications with insufficient evidence of analytical and clinical validity and clinical utility (Khoury et al., 2007; Scheuner et al., 2008).

Current organizations of genetic services in Europe and in selected countries, i.e., the US, Canada, Australia, and New Zealand, form the basis for study by the Personalized PREvention of Chronic Diseases (PRECeDI), a European multicenter project on personalized medicine (Unim et al., 2017; Unim et al., 2019). The project focuses on the transfer of genomic discoveries from research into clinical and public health practice, underlining the barriers and facilitating factors for their implementation and the need for dedicated genomic policies that can support the proper adoption of personalized prevention into healthcare systems. The PRECeDI consortium researched on genetic tests with sufficient evidence of effectiveness and cost-effectiveness, such as tests for hereditary breast and ovarian cancer (HBOC), Lynch syndrome (LS), and familial hypercholesterolemia (FH) (Center of Disease Control and Prevention (CDC)) and on familial thrombophilia (FT), which has insufficient evidence of clinical validity and utility (Evaluation of Genomic Applications in Practice and Prevention (EGAPP) Working Group, 2011; Hickey et al., 2013). Analysis of genetic testing services and associated care pathways identified in the literature enabled the identification and subsequent classification of genetic service delivery models in five categories according to the leading role of healthcare professionals in patient pathways: i) the genetic services led by geneticist model; ii) the primary care model; iii) the medical specialist model; iv) the genetic services integrated into population screening program model; and v) the direct-to-consumer (DTC) model (Unim et al., 2019).

Canada is one of the countries outside of Europe that was included in the PRECeDI multicenter project. The core facilities of genetic services in Canada consist of genetic centers affiliated with universities or healthcare institutions. Professional resources delivering genetic services mainly include genetics staff (e.g., medical geneticists, genetic counselors) and other healthcare providers involved in delivering genetic services as part of multidisciplinary teams (e.g., general practitioners (GPs), medical specialists, nurses, psychologists, and social workers) (Battista et al., 2012). Although there is literature on delivery or organization of genetic services in Canada (Basran et al., 2005; Hanley, 2005; Little et al., 2009; Speechley and Nisker, 2010; Battista et al., 2012; Metcalfe et al., 2012; McCuaig et al., 2018), information regarding the province of Quebec is scarce (Lévesque et al., 2019). In order to provide an evidence base, the present study aims to identify genetic service delivery models for the four selected genetic tests (BRCA1/2, LS, FH, and FT), genomic policies and measures in place to evaluate genetic testing and related services in the province of Quebec.

At the time of the survey, the legislative framework governing the delivery of genetic tests in Quebec and Canada was based on guidelines from local ethics committees, on the voluntary accreditation and participation of genetic laboratories in external quality assessment (EQA) schemes, and on certification of non-medical staff trained in genetics, such as the one provided by the Canadian Association of Genetic Counsellors (CAGC). The Genetic Non-Discrimination Act (GNDA) was adopted by the Canadian Parliament in May 2017, after the collection of the survey data has been completed, and therefore was not addressed in the survey questionnaire. Since then, the Quebec Court of Appeal’s reference decision (December 2018) to the effect that the GNDA is unconstitutional has been appealed to the Supreme Court of Canada and, for now, the Act remains in effect. The GNDA makes a criminal offense to require a person undergoing a genetic test, or obtaining the access to, or forcing someone to disclose the results of such a test as a condition to the provision of goods and services (Government of Canada,). However, it provides exceptions for the use of genetic test results by healthcare professionals and researchers.

## Materials and Methods

### Study Design and Sample Selection

The study was carried out through an online survey available from January to April 2017. The survey targeted healthcare workers, researchers with different backgrounds (e.g., clinical laboratory geneticists, physicians, etc.), and policy makers with good knowledge of and/or practical experience in the provision of at least one of four selected genetic tests (BRCA1/2, LS, FH, and FT), assessment of genetic service delivery models, and policy planning of genetic services in Quebec. To be considered eligible for the study, participants had to be currently practicing in the province. The participants were contacted by email through the Quebec Network of Applied Medical Genetics, the Quebec Association of Genetic Counsellors, and the Canadian Association of Genetic Counsellors. To increase the response rate, participants received an email reminder 6 weeks after the initial invitation email.

### Ethics Statement

Ethics approval for this study was given by McGill University’s Faculty of Medicine Research Ethics Board (study code A01-E02-17A). The informed consent form was administered as the online questionnaire’s first page. The form gave information about the study’s purpose, procedure, risks and benefits of participation, confidentiality, withdrawal, compensation, and contact information of the research coordinators. Participants were informed that there were no foreseeable risks to them and that they could withdraw from the survey at any point. If they chose to participate, their answers could be published in anonymized form. Any identifying elements are only visible to the authors of the study and will be destroyed 5 years after the end of the survey. The recording of informed participant consent was accomplished by participants ticking a checkbox that indicated that they had read the consent form and agreed to participate in the study.

### Questionnaire

The questionnaire items were generated based on literature review and through counseling with European experts in clinical genetics, evaluation of genetic services, and policy making. Further modification of the questionnaire and its translation from English into French was carried out to adapt the tool to the Quebec context.

A bilingual French–English questionnaire was distributed through the online platform Survey Monkey (SurveyMonkey). The first part of the survey [**Supplementary Material (SM 1)**] was on genetic service delivery models for the provision of the four selected genetic tests (BRCA1/2, LS, FT, and FH). It addressed healthcare workers (e.g., medical geneticists, other medical specialists, and genetic counselors) employed in genetic services with manager roles or who were in direct contact with patients requiring one of the aforementioned genetic tests. The survey was composed of the following sections: a) demographic and professional information (five questions, including an open-ended one); and b) genetic service delivery models for BRCA1/2, LS, FT, and FH genetic testing (21 questions, including one optional, open-ended question).

The second part of the survey was on assessment of genetic service delivery models and addressed healthcare workers engaged in health data collection and analysis at local, regional, and provincial levels. It was composed of six sections: a) evaluation of activity (eight questions); b) quality assessment (three questions); c) evaluation of health outcomes (two questions); d) electronic records and genetic information (three questions); and e) genetic services and coverage (two questions, including an optional, open-ended one).

The third part of the survey focused on policy regarding genetic testing and related services; it addressed experts in policy planning/research on genetic services employed in provincial institutions (e.g., national health institute, ministry), universities or clinical research centers. It was composed of the following sections: a) policy (nine questions); b) access and availability of genetic services (eight questions, including an optional, open-ended one); and c) professional education and training (two questions).

### Data Analysis

Descriptive statistics, median, and interquartile range (IQR) were calculated for quantitative variables, while frequencies were generated for qualitative variables. Where possible, the differences between groups of respondents (e.g., physicians, researchers, and policy makers) were calculated with the chi-square test with level of significance set at p < 0.05. The IBM software, Statistical Package for Social Sciences (SPSS) version 25.0 for Windows (SPSS Inc., Chicago, Illinois, USA), was used for data analysis. Two researchers independently analyzed responses to the four open-ended questions. Each researcher identified the items pointed out by the respondents by eliminating and grouping together sub-items in an Excel spreadsheet. Any discrepancies in individual evaluations were resolved through consensus discussion with a third researcher.

## Results

Thirty individuals participated in the study (**Table 1**), with a response rate of 18.75% (30/160). Eighteen respondents completed the questionnaire in French. The healthcare workers were predominantly female (19/30), aged 18–33 (13/30), and genetic counselors (16/30) and had a median of 5 years (IQR 17) of experience in clinical genetics. With regard to clinical practice areas, oncology and oncogenetics (8/16) were the most common. The target populations of healthcare workers consisted mainly of adults, with only three respondents practicing in pediatrics. A majority of respondents have professional experience and/or good knowledge about the provision of BRCA1/2 or LS genetic testing (21/30). Statistically significant differences between the respondents (physicians, genetic counselors, researchers) were observed. In particular, the majority of female respondents are genetic counselors (13/19; p = 0.004), physicians have more years of clinical practice experience (5/5; p = 0.037), and genetic counselors have more experience in the provision of the four genetic tests (6/10; p = 0.015).

**Table 1:** Sociodemographic characteristics of the sample.

VARIABLES	N (%)
***Language***	
English	12 (40%)
French	18 (60%)
	
***Gender***		
F	19 (63.3%)
M	11 (36.7%)
	
***Age***		
18–33	13 (43.3%)
34–49	8 (26.7%)
50–65	5 (16.7%)
> 65 Do not wish to specify	3 (10.0%) 1 (3.3%)
	
***Current position***		
Physician	5 (16.7%)
Genetic counselor	16 (53.3%)
Manager	0 (0)
Researcher	9 (30%)
	
***Medical specialty (N = 16)***		
Medical genetics	3 (18.75%)
Neurogenetics	2 (12.5%)
Oncogenetics	7 (43.75%)
Neurology	3 (18.75%)
Oncology	1 (6.25%)
	
***Years of experience in clinical genetics***		
Median	5 (IQR 17)
	
***Area of clinical practice (N = 16)***		
Medical genetics	3 (18.75%)
Neurology and neurogenetics Oncology and oncogenetics	5 (31.25%) 8 (50.0%)
	
***Professional experience and/or good knowledge of***
BRCA1/2 testing	21 (70%)
Lynch syndrome testing	21 (70%)
Familial thrombophilia	10 (33%)
Familial hypercholesterolemia	11 (36.7%)

F, female; M, male; IQR, interquartile range.

### Genetic Testing: Access to Genetic Services

**Figure 1:**
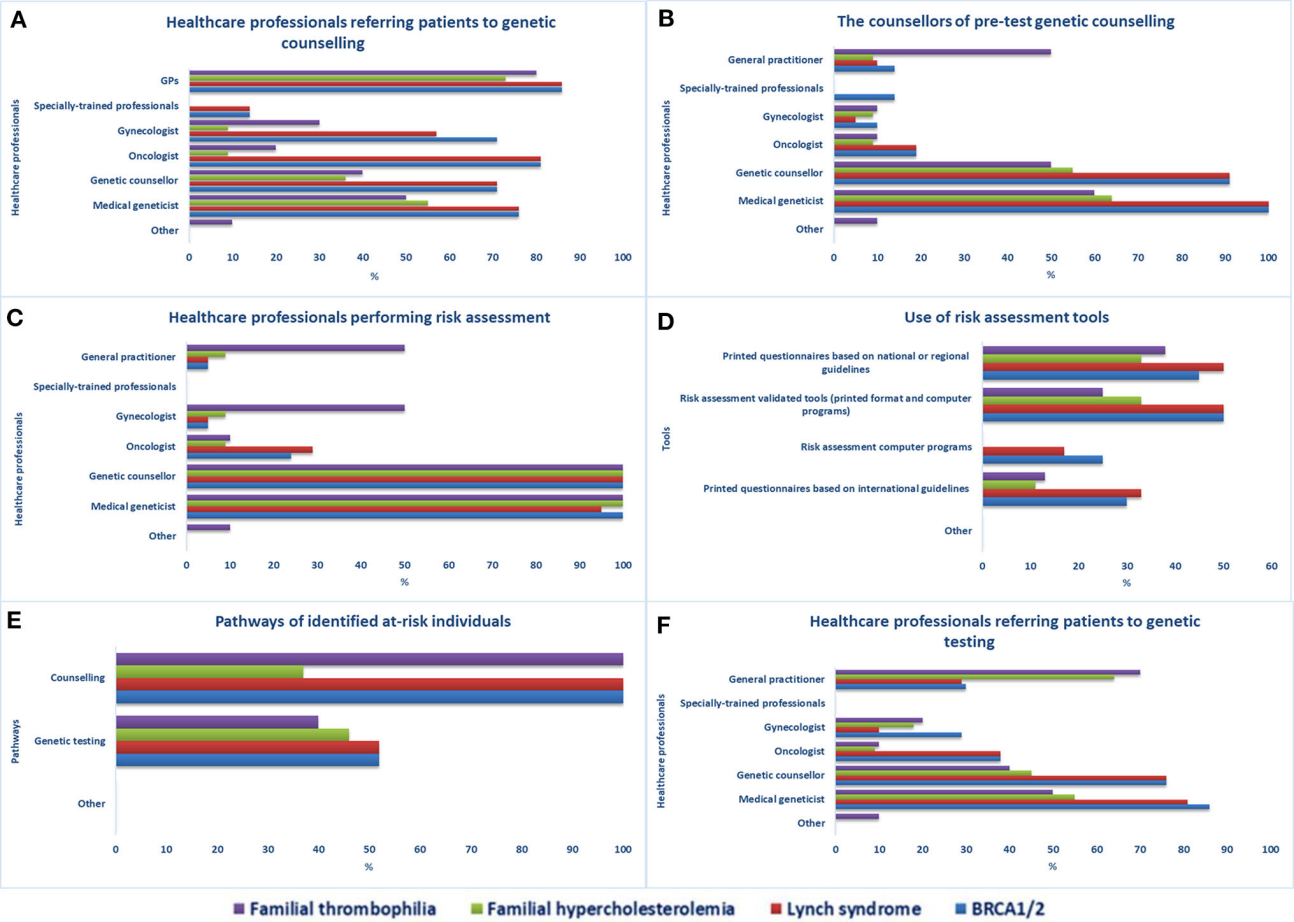
Access to genetic services in Quebec. Healthcare professionals providing **(A)** referrals to genetic counseling services, **(B)** pre-test genetic counseling, and **(C)** performing risk assessment, **(D)** risk assessment tools used in health facilities, **(E)** care pathways of identified at-risk individuals, **(F)** healthcare professionals providing referrals to genetic testing.

Twenty-one participants completed the first part of the survey on genetic testing. They were principally genetic counselors and physicians with a median of 6 years (IQR 18) of experience in clinical genetics. According to respondents, individuals at increased risk of one of the four selected genetic disorders are referred to genetic counseling by various healthcare workers (**Figure 1A**); mostly by GPs, oncologists (BRCA1/2, LS), medical geneticists, genetic counselors, and gynecologists (BRCA1/2, LS, FT). Other access channels to genetic services are direct access (self-referrals) or *via* other medical specialists, nurses, and midwives. Although the counselors of pretest genetic counseling are mainly medical geneticists and genetic counselors (**Figure 1B**), other medical specialists (e.g., GPs, oncologists, cardiologists, gynecologists) and specially trained personnel (e.g., genetic nurses, midwives, physician assistants, etc.) can also provide counseling.

Medical geneticists and genetic counselors are the main healthcare workers performing risk assessment (**Figure 1C**). Other specialists involved are gynecologists and GPs (FT) and cardiologists (FH). According to respondents, specially trained personnel do not perform risk assessment in Quebec. The least-indicated risk assessment tools used in genetic counseling services are computer programs (**Figure 1D**), which are not used at all for the evaluation of individuals at risk of FH and FT. Once identified, individuals at increased risk for one of the four selected genetic disorders are encouraged to undergo genetic counseling (21/21 for the four tests) or genetic testing for BRCA1/2 or LS (11/21), FH (5/11), or FT (4/10) (**Figure 1E**). Various healthcare workers can request genetic testing (**Figure 1F**), mainly medical geneticists, genetic counselors, GPs (FH, FT), oncologists (BRCA1/2, LS), gynecologists (BRCA1/2, FT), and hematologists and cardiologists (FT).

### Pathways After Access to Genetic Testing

**Figure 2:**
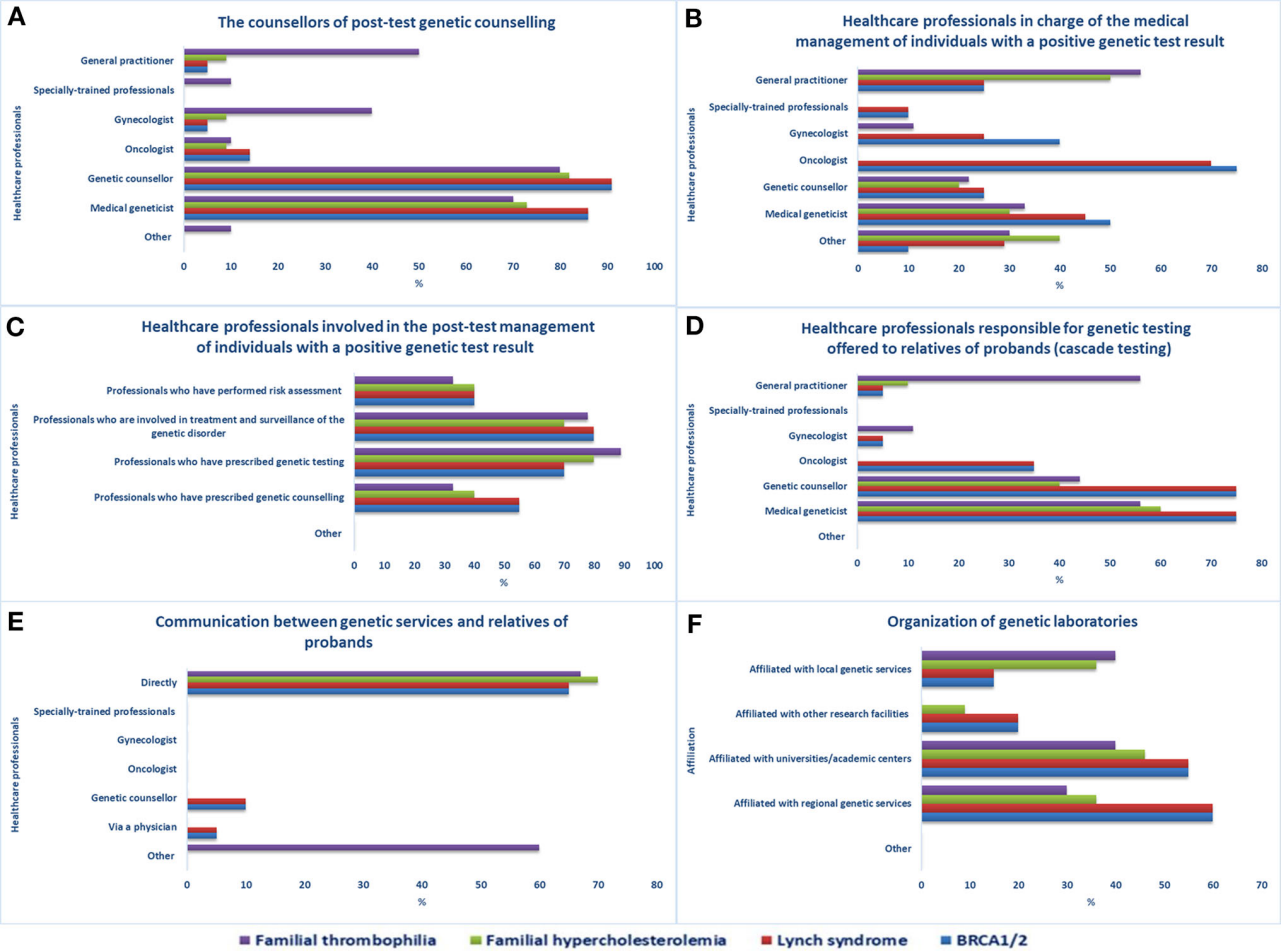
Pathways after access to genetic services. Healthcare professionals **(A)** providing post-test genetic counseling, **(B)** in charge of individuals with a positive genetic test result, **(C)** involved in the post-test management of individuals with a positive genetic test result, **(D)** responsible for genetic testing offered to relatives of probands, **(E)** involved in genetic test results disclosure to relatives of probands, **(F)** organization of genetic laboratories.

Although posttest genetic counseling is mostly performed by genetic counselors, medical geneticists, GPs, and gynecologists (**Figure 2A**), other healthcare workers are also involved (e.g., oncologists, cardiologists, etc.). The clinical management of individuals having a positive BRCA1/2 or LS genetic test (**Figure 2B**) is primarily done by oncologists and medical geneticists, but gastroenterologists (LS), breast surgeons (BRCA1/2), or other medical specialists may also do so, if required. Specially trained personnel, such as care coordinators for BRCA1/2 carriers and research assistants who coordinate and evaluate the follow-up of LS carriers, can also be in charge. GPs, in collaboration with cardiologists, are mainly the ones who handle the clinical care of individuals with a positive FT or FH genetic test. In general, healthcare workers engaged in the care of individuals with a positive genetic test result (**Figure 2C**) are predominantly those involved in treatment and surveillance of the specific genetic disorder and those who prescribed genetic testing.

Genetic testing offered to relatives of probands or index cases (cascade testing) is mostly managed by medical geneticists, genetic counselors, or GPs in the case of FT (**Figure 2D**). After obtaining permission from the proband, staff of an office offering genetic services can inform at-risk relatives of the testing results and invite them to the genetic service for analysis. The means of contact with at-risk relatives (**Figure 2E**) are mostly direct contact (BRCA1/2, LS, FH, FT), proband-mediated contact with or without the provision of a family letter or other written information (FT), or any form of physician-mediated contact (BRCA1/2, LS). According to the majority of the sample, referring healthcare workers are always informed about the patient’s genetic test results (15/21) and genetic laboratories participate in quality control procedures (17/21). The laboratories are mostly affiliated with regional genetic services (BRCA1/2, LS, FH), academic centers (BRCA1/2, LS, FH, FT), or local genetic services (FH, FT) (**Figure 2F**).

### Genetic Service Delivery Models

Medical geneticists have the most prominent role in the provision of genetic tests, the coordination of treatment, and the monitoring of patients by a multidisciplinary team. However, some respondents specified that the decision regarding who takes on the principal role in care is made on a case-by-case basis according to the underlying genetic disorder. For instance, oncologists have the most prominent role in the case of BRCA1/2 or LS and cardiologists in the case of FT. GPs and other medical specialists were indicated as professional figures with a prominent role in genetic test provision and the monitoring of patients undergoing FT and FH testing. For BRCA1/2 and LS testing, respondents indicated that prominent roles were assumed by physicians engaged in population screening programs.

The delivery models for the provision of the four genetic tests in Quebec are classified in five categories (**Table 2**) according to the healthcare personnel with the most prominent role in genetic test provision, treatment and monitoring of patients, and the care pathways (i.e., a patient’s path through different healthcare personnel “from the initial point of access to healthcare services to treatment of the genetic disorder and follow-up”) (Unim et al., 2019). The delivery models are described below.

**Table 2:** |Genetic service delivery models for the provision of predictive genetic testing in Quebec.

PATHWAY	Model I: Genetic services led by geneticists	Model II: Primary Care Model	Model III: Medical Specialist Model	Model IV: Genetic services integrated into population screening programs	Model V: Direct to consumer (DTC)
**A**	Patient → GP or medical specialist → Counselor^b^→ Lab	Patient → GP → Counselor → Lab	Patient → Medical specialist → Lab	Patient → GP or medical specialist → Counselor → Lab	Patient → Lab
**B**	Patient → Counselor (Medical specialist) → Lab	Patient → GP → Lab	Patient → Medical specialist → Counselor → Lab	Patient → GP or medical specialist → Lab	
**C**				Patient → Counselor → Lab	
**TESTING**	BRCA1/2, LS, FH, FT	BRCA1/2, LS, **FH, FT**	BRCA1/2, LS, **FH, FT**	BRCA1/2, LS	Various

GP, General practitioner; Counselor, counseling could be provided by geneticists or genetic counselors; LS, Lynch syndrome; FH, familial hypercholesterolemia; FT, familial thrombophilia; in bold, main genetic tests provided under a specific model.

Model I: Genetic services led by geneticists. The genetic team may include medical geneticists, genetic counselors, and other healthcare workers (e.g., genetic nurses). The genetic team is responsible for risk assessment, counseling, and testing of individuals or families affected by or at risk for genetic disorders. Depending on the case, the team collaborates with other medical specialists (e.g., oncologists, cardiologists, nephrologists, etc.) who could be part of the genetic service (e.g., multidisciplinary genetic clinics). The access of patients to this model of genetic services may occur through two different pathways: a) Patient → GP or medical specialist → Counselor → Lab (Unim et al., 2019); and b) Patient → Counselor (medical specialists) → Lab. All four selected genetic tests are provided under this model, but BRCA1/2 and LS genetic tests predominate.

Model II: Primary care model. A prominent role is played by primary care units, which may include primary care physicians (GPs or family physicians), a nurse practitioner, or a physician assistant. In these units, GPs have some training in genetics and can undertake an initial risk assessment using standardized referral guidelines. In some cases, GPs refer at-risk patients to genetic services, while in other cases, they care for patients without consulting medical geneticists or genetic counselors. The pathways associated to this model are: a) Patient → GP → Lab and b) Patient → GP → Counselor → Lab (Unim et al., 2019). Under this model of delivery, FH and FT genetic tests are the most frequently provided.

Model III: Medical specialist model. Genetic tests can be requested directly by medical specialists (e.g., oncologists, cardiologists, neurologists, etc.) who may be able to manage patients with or at risk of genetic disorders without consulting medical geneticists or genetic counselors. The possible pathways in Model III are: a) Patient → Medical specialist → Lab and b) Patient → Medical specialist → Counselor → Lab (Unim et al., 2019). The four genetic tests are provided under this model, but mostly FH and FT tests.

Model IV: Genetic services integrated into population screening programs. In this model, genetic services are provided within organized population screening programs (e.g., HBOC screening, colorectal cancer screening, population-based screening of Ashkenazi Jews). There are three possible patient pathways in Model IV: a) Patient → GP or medical specialist → Counselor → Lab; b) Patient → GP or medical specialist → Lab; and c) Patient → Counselor → Lab. The IVa pathway occurs when a patient takes part in a population-based screening program; a healthcare professional involved in the screening program can perform an initial risk assessment and refer the patient to genetic counseling. The genetic counselor or medical geneticist can suggest genetic testing and, based on results of the test, can make recommendations as to monitoring and/or clinical intervention. In the IVb pathway, healthcare personnel involved in a population-based screening program can perform risk assessment and suggest genetic testing. Based on results of the test, the healthcare personnel can make recommendations as to monitoring and/or clinical intervention. In the IVc pathway, a patient contacts a genetic counselor or a medical geneticist who can suggest genetic testing and, based on results of the test, can recommend monitoring through available population-based screening programs and/or clinical intervention (Unim et al., 2019). Only BRCA1/2 and LS genetic tests are provided under this model.

In Model V: DTC model, genetic testing services are offered online by private companies. Healthcare professionals are not usually involved in the process, nor are medical referrals required for genetic testing. The main pathway associated with Model V is Patient → Lab (Unim et al., 2019). Although the DTC model was not indicated as an available pathway within the health facilities, seven respondents acknowledged its presence in the province and declared that patients interested in DTC genetic testing for any genetic disorder may receive counseling and follow-up services in their health facilities.

The implementation of genetic services for predictive genetic testing has resulted in the development of new roles, namely, genetic nurses and genetic care coordinators. The main factors that led to the current genetic service delivery models in Quebec (**Table 3**) are “availability of specialized staff/qualified personnel,” “availability of specialized centers and laboratories,” and “coverage of genetic tests/test offer.” The availability of specialized or qualified personnel was the most frequently indicated factor for the four genetic tests. Barriers for the current genetic service delivery models are predominantly “lack of professional resources in medical genetics (e.g., genetic counselors, medical geneticists),” “access to genetic services/patients not referred to genetic services,” and “inadequate number or distribution of counseling centers in Quebec.” Lack of professional resources in medical genetics is the most frequently cited critical issue for the four genetic tests.

**Table 3:** Barriers and facilitating factors of the currently available genetic service delivery models in Quebec.

		NUMBER OF TIMES THE ITEM WAS MENTIONED				
	ITEMS		BRCA	LYNCH	FT	FH
*BARRIERS*	Lack of professional resources in medical genetics (e.g., genetic counselors, medical geneticists)		8	9	5	4
	Access to genetic services/patients not referred to genetic services		3	3	–	–
	Inadequate number or distribution of counseling centers in Quebec		3	3	–	–
	Insufficient training courses in genetics for general practitioners		2	2	1	1
	Administrative slowness		2	1	1	1
	Lack of knowledge on genetic diseases (i.e., in primary care)		2	1	–	–
	Service organization		2	1	–	1
	Tests done outside the costly province		1	–	–	–
	Lack of laboratory equipment		1	1	1	1
	Low use of modern technologies (PC, internet, electronic records, etc.)		2	2	–	–
*FACILITATING* *FACTORS*	Availability of specialized staff/qualified personnel		10	11	5	6
	Availability of specialized centers and laboratories		3	3	1	2
	Coverage of genetic tests/test offer		3	3	1	1
	Appropriate requests for consultation by the family doctors/specialists		2	2	–	–
	Research		1	1	1	1

Lynch, Lynch syndrome; FH, familial hypercholesterolemia; FT, familial thrombophilia.

### Assessment of Genetic Services

**Figure 3:**
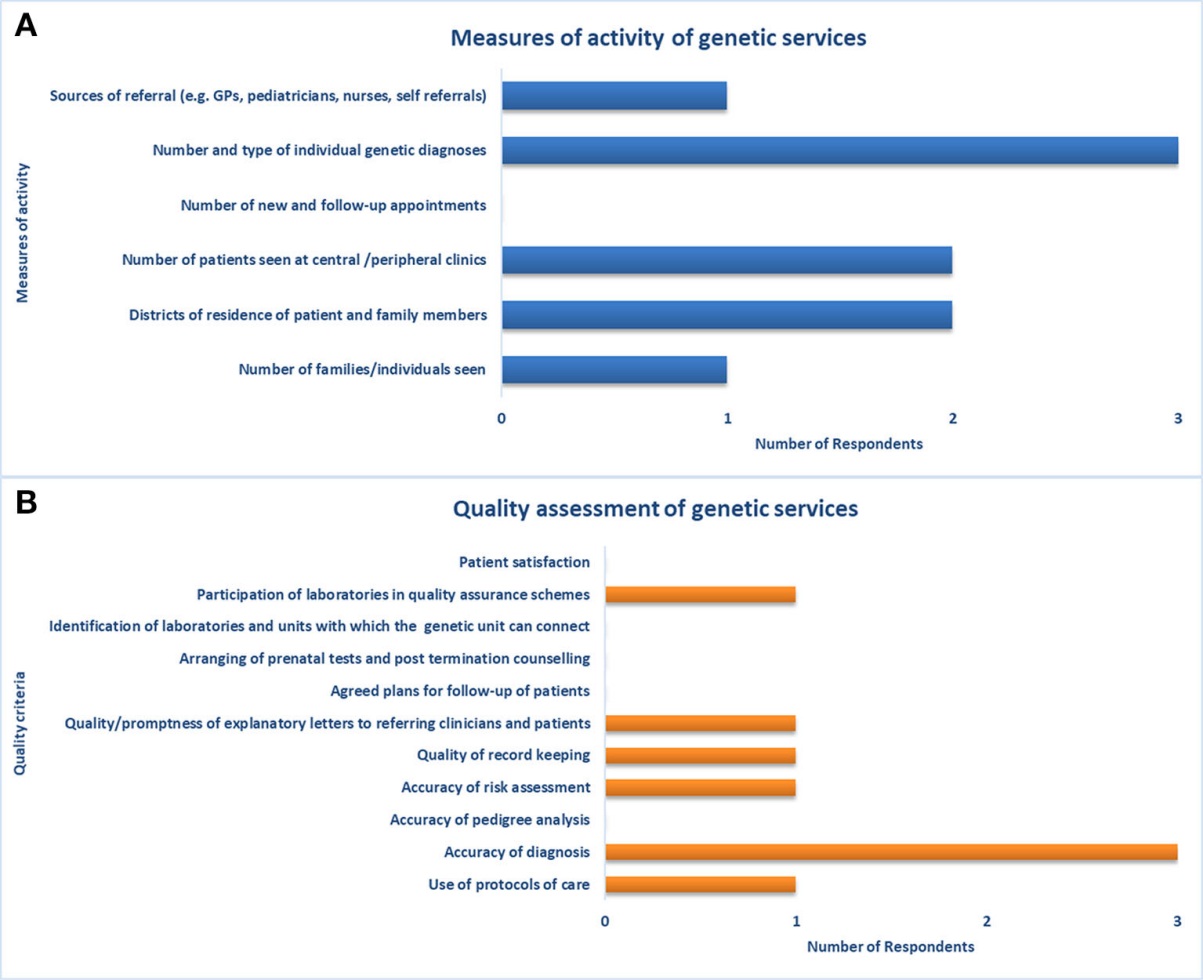
Measures of activity and quality assessment of genetic services. Indicators used for assessment of **(A)** capacity of and access to genetic services, and **(B)** quality of genetic services.

Three participants with expertise in health data collection and analysis completed the second part of the survey on the assessment of genetic services. The respondents consisted of two physicians and a genetic counselor with a median of 20 years (IQR not computed) of experience in clinical genetics. According to these respondents, two health facilities usually collect, store, and retrieve data on genetic services through electronic processes. Despite the adoption of electronic processes, a link to provincial-level patient data is only available in one facility. Among the measures of activity of genetic services available in Quebec, the respondents did not indicate the number of new and follow-up appointments (**Figure 3A**). Moreover, they did not report on several indicators among quality assessment measures (e.g., accuracy of pedigree analysis, patient satisfaction, patient-reported outcomes, etc.) and outcome measures (e.g., morbidity and mortality rates) (**Figure 3B**).

The respondents declared that electronic records currently implemented in Quebec [Dossier Santé Québec (DSQ)] include genetic information but were not aware of the possibility to use DSQ information to evaluate the appropriateness of care according to a patient’s condition or diagnosis. The genetic conditions that are met with an adequate provision of genetic services in Quebec, as indicated by the respondents, are BRCA1/2, cardiovascular diseases, Huntington disease, and neurological conditions (e.g., Alzheimer, Parkinson).

### Policies Governing the Provision of Genetic Services

Of 30 respondents, 26 completed the third part of the survey on policies of genetic services. Three respondents are fully engaged in policy planning and/or research on genetic services, who are physicians (medical geneticists, neurologists) and researchers with a median of 20 years (IQR not computed) of experience in clinical genetics. More researchers (8/16; p = 0.002) are aware of guidelines from local ethics committees for the evaluation of research protocols involving biobanks. However, few specified that the guidelines originate from the Tri-Council Policy Statement. Genetic counselors (8/12; p = 0.04) are more aware that, in Quebec, the accreditation and participation of genetic laboratories in EQA schemes are not mandatory but are promoted. Most respondents are aware of legislations governing the practice of non-medical health professionals, such as genetic nurses and technical staff trained in genetics (15/26). The certification system in Quebec for non-medical staff trained in genetics is also well known by the respondents (21/26), of which seven indicated the CAGC and the American Board of Genetic Counselling (ABGC). The respondents declared that no specific legislation addresses DTC genetic testing. They indicated that provincial or local guidelines that could help health departments organize genetic services to serve also as research and educational resources are currently under development.

**Figure 4:**
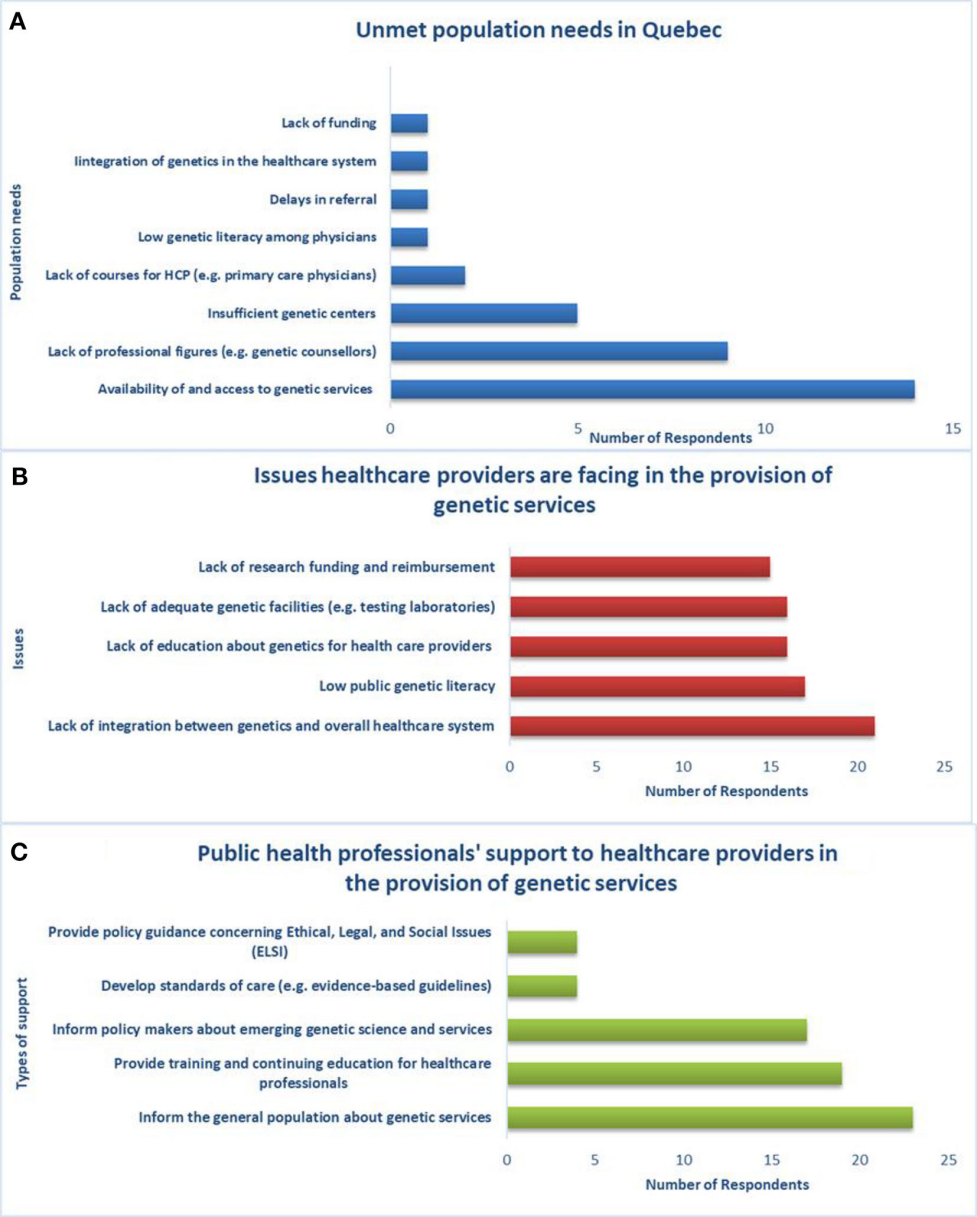
Unmet population needs, healthcare providers’ issues and support from public health professionals, **(A)** population needs are not met by the current provision of genetic services in Quebec, **(B)** there is lack of integration between genetics and the overall healthcare system, **(C)** public health professionals can support genetic service providers in several ways.

Regarding access and availability of genetic services, genetic laboratories are mostly in the public sector (23/26) and public health insurance covers genetic tests of proven efficacy and related services (e.g., BRCA1/2 predictive testing, bilateral preventive mastectomy, preventive salpingo-oophorectomy) (26/26). Generally, genetic tests requested by physicians according to clinical guidelines are accepted in health facilities across the province and are covered by public health insurance. However, the current provision of genetic services does not adequately meet the population’s needs in Quebec according to the majority of the sample (25/26; p = 0.038). Key needs include the availability of and access to genetic services, qualified personnel (e.g., genetic counselors), and genetic centers (**Figure 4A**). New approaches, such as telemedicine, are under development to meet the demand for genetic services of underserved populations.

The main issue that healthcare workers (physicians, nurses, laboratory staff, genetic counselors, etc.) are facing with respect to the provision of genetic services in Quebec (**Figure 4B**) is lack of integration between genetics and the greater healthcare system (21/26). Respondents indicated that public health professionals can support healthcare providers in the provision of genetic services (**Figure 4C**) by informing the general population about genetic services (23/26), providing training and continuing education for healthcare workers (19/26), and informing policy makers about emerging genetic science and services (17/26). With regard to education and training, the respondents are aware of courses in PHG provided to genetic counselors (26/26), physicians (24/26), nurses (19/26), and lab technicians (10/26).

## Discussion

In order to describe the different aspects of genetic service provision and its related issues in the second most populous Canadian province, we present survey responses by 30 healthcare personnel who are currently involved in clinical practice, research, or policy making concerning genetic services in Quebec. The findings of the present study confirm the genetic service delivery models identified through a previous literature review (Unim et al., 2019). Model I: Genetic service led by geneticists is the most common model of genetic service provision for the four genetic tests, especially for BRCA1/2 and LS testing. In this model, medical geneticists have a prominent role in care pathways and coordinate the treatment and monitoring of patients in a multidisciplinary team. However, other medical specialists (e.g., GPs, cardiologists, gynecologists, oncologists, etc.) are increasingly involved in delivering genetic services as part of multidisciplinary teams and can also assume a prominent role in patient care according to the genetic disorder. This aspect confirms the existence of other models of delivery in which various specialists are able to care for patients with or without consulting medical geneticists.

Indeed, the four genetic tests are also provided under Model II: Primary Care Model and Model III: Medical Specialist Model, while BRCA1/2 and LS genetic tests are also offered under Model IV: Genetic services integrated into population screening programs. The gradual integration of medical genetics in other disciplines has led to a major involvement of various healthcare professionals in genetic service provision and has paved the way for the development of new roles (e.g., genetic nurses, genetic counselors, genetic care coordinators) that actively support genetic teams in Quebec, as in other settings (Allen et al., 2007; Bennett et al., 2010; Burton et al., 2010; Bell et al., 2015; Unim et al., 2019). This is an attempt to adapt the healthcare system to the rapid development of genomic technologies that cannot be sustained by only one professional figure in the long run and requires collaboration among healthcare personnel and the redistribution of work. Nonetheless, the development of genetic service delivery models should not be entirely based upon the creation of new professional roles because the full beneficial effects of genetic medicine will not be felt for many years. Efforts are better directed toward enhancing the ability of currently available professional resources.

There were no differences in the delivery of genetic tests with considerable evidence of efficacy and cost-effectiveness (i.e., BRCA1/2, LS, and FH) (Center of Disease Control and Prevention (CDC)) when compared to FT genetic test, which has insufficient evidence of clinical utility and validity (Evaluation of Genomic Applications in Practice and Prevention (EGAPP) Working Group, 2011; Hickey et al., 2013). In fact, FT genetic test is provided to the general population by geneticists and other medical specialists, mostly gynecologists, GPs, hematologists, and cardiologists. “Prior to implementation in clinical and public health practice, genetic tests should be evaluated based on available data as to their efficacy and cost-effectiveness. Only those tests with proven efficacy and cost-effectiveness should be implemented in clinical and public health practice” (Unim et al., 2019). Moreover, according to the three respondents who answered the second section of the survey, some quality and outcome indicators are not collected or used to assess the appropriateness of genetic procedures in the province. Although these findings cannot be generalized given the low response rate, clinical pathways should be monitored to reduce inappropriate provision of genetic services and to ensure high quality standards of the services.

Most facilitating factors for the implementation of genetic service delivery models, such as the provision of genetic tests, availability of qualified personnel, and specialized centers, are also identified as the barriers for the appropriate provision of genetic services in Quebec, which seems counterintuitive. However, this indicates that the rate of genetic specialists and specialized centers have not kept pace with the increase of genetic discoveries and the related increase in demand for genetic testing and counseling services in the general population. Regarding the provision of genetic tests, new models of delivery have to be devised within the healthcare system to enhance the access to genetic testing. The integration of genetic services into population screening programs (e.g., for breast or colon cancer) is a justifiable option and complementary to the more established model led by geneticists. It uses already available services and healthcare providers to enhance referrals to genetic testing, which in turn facilitates the identification and follow-up of those at high risk of inherited disorders. Unfortunately, it is a relatively new model of delivery and not yet widely implemented. Among Canadian provinces, the integration between screening and genetic testing services is under development in Quebec and guidelines are implemented only in the context of breast cancer screening programs in Alberta (Alberta Health Services, 2015), British Columbia (British Columbia Guidelines, 2013), and Ontario (Cancer Care Ontario, 2015). The development of initiatives to promote access to medical genetics services in remote areas is also necessary to guarantee adequate coverage of genetic services in the province of Quebec (Lévesque et al., 2019).

The barriers and facilitating factors indicated by the respondents are related not only to the number of qualified personnel or centers but also to healthcare providers' knowledge and skills in medical genetics. A respondent summarized this issue as: “*Too many non-geneticists claim to be geneticists, too many professionals do genetic counseling without proper training.”* In light of this, policies aiming at the improvement of undergraduate and postgraduate training in medical genetics, including ethics, are of paramount importance to enhance knowledge and competency of various healthcare workers in providing genetic services. Certifications of national associations, such as those provided by the CAGC and the ABGC, are equally important to guarantee the proper provision of genetic services by qualified healthcare providers. Indeed, standards defining training, practice, and registration requirements for genetic counselors have been developed in Canada by the CAGC and in the US by the ABGC (Ferrier et al., 2013). Core competencies for genetic counselors have also been defined in Europe (Ferrier et al., 2013; Paneque et al., 2016) and Australia (Sahhar et al., 2005). The adoption of practice standards can harmonize the differences between countries in genetic counseling education and practice (Ferrier et al., 2013; Paneque et al., 2016).

Although a national certification is available, the genetic counseling profession is currently unregulated in Canada; hence, genetic counselors are not governed by national or provincial legislation that regulates the practice of other non-medical healthcare professionals, such as nurses (The Canadian Association of Genetic Counsellors (CAGC)). This issue is not unique to Canada—the lack of national or provincial regulation governing the practice of genetic counselors is a common issue worldwide. National regulations currently only exist in the UK, Norway, Israel, Saudi Arabia, and South Africa, with projects to implement national regulations underway in Australia and New Zealand. A state-level regulation exists in only 22 (of 50) states in the US (Abacan et al., 2019). There is a need for a unified approach for the appropriate regulation of quality and competence in genetic service provision. The lack of specific regulatory oversight concerning genetic testing in Canada includes the provision of genetic services over the internet (DTC model) (Melzer et al., 2008; Fukuda and Takada, 2018). This is certainly a major gap in policy regulations that needs to be swiftly addressed given the critical issues related to the provision of DTC services, such as lack of access to qualified counseling and proper interpretation of test results to better understand their implications for the individual.

### Limitations

The study was limited by the small number of respondents and high item nonresponse rate prevalent in the section regarding assessment of genetic services. These may reflect the limitations inherent in the provision of genetic services in Quebec. However, the respondents are healthcare personnel with good knowledge of and many years of experience in the provision of genetic testing services in the province. It should be also noted that online surveys have an average response rate ranging between 20% and 30% (Safdar et al., 2016), and surveys conducted among physicians and other healthcare personnel have about 10% lower response rates than general population surveys (Cummings et al., 2001). The response rate of the present study is in accordance with these findings, and the final sample size was sufficient to complete the descriptive analysis and to gain an in-depth understanding of genetic service organizational structure in the province of Quebec by acquiring information from highly qualified personnel in the field of clinical genetics. The three sections of the questionnaire enabled the collection of opinions on genetic service delivery models in terms of strengths, weaknesses, and possible improvements of the existing models. The study was also limited by the cross-sectional design, which is common to all surveys, that could lead to misinterpretation of the questions, underreporting, and recall bias. However, in the absence of an interviewer, respondents may be more prone to share information and provide more truthful responses when anonymity and confidentiality are guaranteed (Safdar et al., 2016).

## Conclusions

Currently, there are few legislative frameworks in Quebec and Canada that specifically target genetic testing and related services. The provision of genetic testing is based on guidelines from local ethics committees, voluntary participation in EQA schemes, standards of practice developed by the CAGC for genetic counselors, and the recent GNDA. The Act aims at preventing genetic discrimination by introducing a criminal prohibition to require genetic testing or, obtaining the access to, or force to disclose information obtained through genetic testing with regard to the provision of goods and services. The Act also prohibits the collection, use, and disclosure of genetic test results without a written consent. The GNDA provides an exception for physicians providing health services and researchers.

The study highlighted that the integration of genetics and the greater healthcare system in Quebec is still at an early phase. There is therefore a critical need for an integration of knowledge in genomic medicine within and across multiple disciplines to enhance the use of existing and new genomic applications. A multidisciplinary team composed of physicians with different specialties and public health professionals should provide training to improve the identification of at-risk individuals, patient referrals to testing, selection of appropriate tests, and interpretation of the test results. Training and education of healthcare personnel in genetics could be improved by direct supervision, encouraging collaborations between healthcare practitioners with different backgrounds and roles to increase the interactions between health providers, improve the detection of disorders with a genetic basis, and achieve better teamwork. Certification of non-medical staff trained in genetics through national (e.g., ABCG, CAGC) and international (e.g., European Board of Medical Genetics) associations should be mandatory.

Genomic applications need to be evaluated before integrating genomic information into the healthcare system. The implementation of genetic discoveries should be governed by appropriate legislative frameworks that can ensure quality by setting standards, evaluating performance, and monitoring outcomes of services. In light of this, a global approach involving national and international professional organizations working with government agencies is necessary to ensure that genetic testing and related services are of a high quality and consumers are protected.

In conclusion, current delivery models require good level of genetic knowledge, adequate funding, public policies, and public understanding of genetics and genomics applications. Moreover, different healthcare personnel need to take responsibility for the provision of genetic services to guarantee the sustainability of the genetic service delivery models.

To our knowledge, this is the first study that identifies and classifies genetic service delivery models in Quebec. The study has highlighted elements of good practice in genetic service provision and areas for improvement of current genetic service delivery models that may be of use to healthcare providers and policy makers actively involved in personalized medicine.

## Data Availability Statement

The datasets generated for this study will not be made publicly available because they include information that could compromise research participant privacy/consent.

## Ethics Statement

The studies involving human participants were reviewed and approved by McGill University’s Faculty of Medicine Research Ethics Board (study code A01-E02-17A). The patients/participants provided their written informed consent to participate in this study.

## Author Contributions

All authors contributed to the conception and design of the study. BU, JH, MZ, and BK reviewed and translated the survey instrument. BU organized the database. BU and CV performed the statistical analysis. BU and PV wrote the manuscript with input from all authors. All authors read and approved the final manuscript.

## Funding

The study is funded by the PRECeDI project (Marie Sklodowska-Curie Research and Innovation Staff Exchange-RISE), Grant agreement no: 645740.

## Conflict of Interest

The authors declare that the research was conducted in the absence of any commercial or financial relationships that could be construed as a potential conflict of interest.
