# Supplementary material for: The Provision of Genetic Testing and Related Services in Quebec, Canada
Source: Front Genet. 2020 Mar 4;11:127. doi: 10.3389/fgene.2020.00127 (PMC7064617; doi:10.3389/fgene.2020.00127)
Supplement: Supplementary file 1 [file DataSheet_1.pdf]

Supplementary Material 1

Questionnaire: Identification of delivery models for the provision of predictive genetic testing in the province of Quebec

Dipartimento Sanità Pubblica e Malattie Infettive

SAPIENZA UNIVERSITÀ DI ROMA

McGill

CGP Centre of Genomics and Policy
Centre de génomique et politiques

IDENTIFICATION OF DELIVERY MODELS FOR THE PROVISION OF PREDICTIVE GENETIC TESTING IN THE PROVINCE OF QUEBEC

DEMOGRAPHIC AND PROFESSIONAL INFORMATION

In this section you are kindly asked to provide some personal information.

* 1. GENDER

- ○ Male
- ○ Female
- ○ Do not wish to specify

* 2. AGE (years)

- ○ 18-33
- ○ 34-49
- ○ 50-65
- ○ >65
- ○ Do not wish to specify

* 3. CURRENT POSITION (select one or more answers)

- ☐ Physician
- ☐ Genetic counsellor
- ☐ Manager
- ☐ Researcher
- ☐ Do not wish to specify
- ☐ Other (please specify)

Please specify the medical specialty, if applicable

* 4. HOW MANY YEARS OF TOTAL WORK EXPERIENCE IN CLINICAL GENETICS DO YOU HAVE?

* 5. DO YOU HAVE PROFESSIONAL EXPERIENCE AND/OR GOOD KNOWLEDGE OF THE PROVISION OF THE FOLLOWING GENETIC TESTS IN THE PROVINCE OF QUEBEC? (Select one or more answers)

	Yes	No
BRCA1/2	○	○
LYNCH SYNDROME	○	○
FAMILIAL THROMBOPHILIA	○	○
FAMILIAL HYPERCHOLESTEROLEMIA	○	○

Dipartimento Sanità Pubblica e Malattie Infettive

CGP Centre of Genomics and Policy / Centre de génomique et politiques

IDENTIFICATION OF DELIVERY MODELS FOR THE PROVISION OF PREDICTIVE GENETIC TESTING IN THE PROVINCE OF QUEBEC

PART 1. GENETIC TESTING

In this section you are kindly asked to answer questions according to your professional experience and/or good knowledge on genetic service delivery models **for the provision of BRCA1/2, Lynch syndrome, Familial Thrombophilia or Familial Hypercholesterolemia genetic testing and the associated patients' pathways to care in your institution.**

Genetic service delivery models can be defined as the broad context within the Public Health Genomics framework in which genetic tests are offered to individuals and families with or at risk of genetic disorders.

A. ACCESS TO GENETIC TESTING

* 1. WHO CAN REFER TO GENETIC COUNSELLING IN YOUR INSTITUTION? (Select one or more answers for each genetic test)

	General practitioner	Medical geneticist	Genetic counsellor	Oncologist	Gynecologist	Specially-trained professionals	Other
BRCA1/2	☐	☐	☐	☐	☐	☐	☐
LYNCH SYNDROME	☐	☐	☐	☐	☐	☐	☐
FAMILIAL THROMBOPHILIA	☐	☐	☐	☐	☐	☐	☐
FAMILIAL HYPERCHOLESTEROLEMIA	☐	☐	☐	☐	☐	☐	☐

Other (please specify)

[ ]

Please specify the specially-trained professionals, if applicable (e.g. genetic nurses, midwives, physician assistants)

[ ]

* 2. WHO ARE THE COUNSELLORS OF PRE-TEST GENETIC COUNSELLING IN YOUR INSTITUTION? (Select one or more answers for each genetic test)

	General practitioner	Medical geneticist	Genetic counsellor	Oncologist	Gynecologist	Specially-trained professionals	Other
BRCA1/2	☐	☐	☐	☐	☐	☐	☐
LYNCH SYNDROME	☐	☐	☐	☐	☐	☐	☐
FAMILIAL THROMBOPHILIA	☐	☐	☐	☐	☐	☐	☐
FAMILIAL HYPERCHOLESTEROLEMIA	☐	☐	☐	☐	☐	☐	☐

Other (please specify)

Please specify the specially-trained professionals, if applicable (e.g. genetic nurses, midwives, physician assistants)

* 3. WHO CAN REFER DIRECTLY TO GENETIC TESTING IN YOUR INSTITUTION? (Select one or more answers for each genetic test)

	General practitioner	Medical geneticist	Genetic counsellor	Oncologist	Gynecologist	Specially-trained professionals	Other
BRCA1/2	☐	☐	☐	☐	☐	☐	☐
LYNCH SYNDROME	☐	☐	☐	☐	☐	☐	☐
FAMILIAL THROMBOPHILIA	☐	☐	☐	☐	☐	☐	☐
FAMILIAL HYPERCHOLESTEROLEMIA	☐	☐	☐	☐	☐	☐	☐

Other (please specify)

Please specify the specially-trained professionals, if applicable (e.g. genetic nurses, midwives, physician assistants)

* 4. WHO CAN PERFORM RISK ASSESSMENT IN YOUR INSTITUTION? (Select one or more answers for each genetic test)

	General practitioner	Medical geneticist	Genetic counsellor	Oncologist	Gynecologist	Specially-trained professionals	Other
BRCA1/2	☐	☐	☐	☐	☐	☐	☐
LYNCH SYNDROME	☐	☐	☐	☐	☐	☐	☐
FAMILIAL THROMBOPHILIA	☐	☐	☐	☐	☐	☐	☐
FAMILIAL HYPERCHOLESTEROLEMIA	☐	☐	☐	☐	☐	☐	☐

Other (please specify)

____

Please specify the specially-trained professionals, if applicable (e.g. genetic nurses, midwives, physician assistants)

____

* 5. WHAT TYPES OF TOOLS ARE USUALLY USED FOR RISK ASSESSMENT IN YOUR INSTITUTION? (Select one or more answers for each genetic test)

	Questionnaires based on national or regional guidelines (printed format)	Questionnaires based on international guidelines (printed format)	Risk assessment computer programs	Risk assessment validated tools (printed format and computer programs)	Other
BRCA1/2	☐	☐	☐	☐	☐
LYNCH SYNDROME	☐	☐	☐	☐	☐
FAMILIAL THROMBOPHILIA	☐	☐	☐	☐	☐
FAMILIAL HYPERCHOLESTEROLEMIA	☐	☐	☐	☐	☐

Other (please specify)

____

* 6. ONCE IDENTIFIED, AT-RISK INDIVIDUALS IN YOUR INSTITUTION ARE ENCOURAGED TO UNDERGO (Select one or more answers for each genetic test):

	Genetic counselling	Genetic testing	Other
BRCA1/2	☐	☐	☐
LYNCH SYNDROME	☐	☐	☐
FAMILIAL THROMBOPHILIA	☐	☐	☐
FAMILIAL HYPERCHOLESTEROLEMIA	☐	☐	☐

Other (please specify)

______

B. PATHWAYS AFTER ACCESS TO GENETIC TESTING

* 1. HOW ARE GENETIC LABORATORIES ORGANIZED IN YOUR INSTITUTION? (Select one or more answers for each genetic test)

	Affiliated with local genetic services	Affiliated with regional genetic services	Affiliated with universities/academic centers	Affiliated with other research facilities	Other
BRCA1/2	☐	☐	☐	☐	☐
LYNCH SYNDROME	☐	☐	☐	☐	☐
FAMILIAL THROMBOPHILIA	☐	☐	☐	☐	☐
FAMILIAL HYPERCHOLESTEROLEMIA	☐	☐	☐	☐	☐

Other (please specify)

______

Please specify "other research facilities", if applicable

______

* 2. DO GENETIC LABORATORIES IN YOUR INSTITUTION PARTICIPATE IN QUALITY CONTROL PROCEDURES? (Select one answer for each genetic test)

	Yes	No
BRCA1/2	○	○
LYNCH SYNDROME	○	○
FAMILIAL THROMBOPHILIA	○	○
FAMILIAL HYPERCHOLESTEROLEMIA	○	○

* 3. WHO ARE THE COUNSELLORS OF POST-TEST GENETIC COUNSELLING IN YOUR INSTITUTION? (Select one or more answers for each genetic test)

	General practitioner	Medical geneticist	Genetic counsellor	Oncologist	Gynecologist	Specially-trained professionals	Other
BRCA1/2	☐	☐	☐	☐	☐	☐	☐
LYNCH SYNDROME	☐	☐	☐	☐	☐	☐	☐
FAMILIAL THROMBOPHILIA	☐	☐	☐	☐	☐	☐	☐
FAMILIAL HYPERCHOLESTEROLEMIA	☐	☐	☐	☐	☐	☐	☐

Other (please specify)

Please specify the specially trained professionals, if applicable (e.g. genetic nurse, midwives, physician assistants)

* 4. ARE REFERRING HEALTH CARE PROFESSIONALS IN YOUR INSTITUTION INFORMED ABOUT THE GENETIC TESTING RESULTS OF THE TESTEES? (Select one answer for each genetic test)

	Yes, always	Yes, sometimes	No
BRCA1/2	○	○	○
LYNCH SYNDROME	○	○	○
FAMILIAL THROMBOPHILIA	○	○	○
FAMILIAL HYPERCHOLESTEROLEMIA	○	○	○

* 5. WHO TAKES CHARGE OF THE MEDICAL MANAGEMENT OF INDIVIDUALS HAVING A POSITIVE GENETIC TEST RESULT IN YOUR INSTITUTION? (Select one or more answers for each genetic test)

	General practitioner	Medical geneticist	Genetic counsellor	Oncologist	Gynecologist	Specially-trained professionals	Other
BRCA1/2	☐	☐	☐	☐	☐	☐	☐
LYNCH SYNDROME	☐	☐	☐	☐	☐	☐	☐
FAMILIAL THROMBOPHILIA	☐	☐	☐	☐	☐	☐	☐
FAMILIAL HYPERCHOLESTEROLEMIA	☐	☐	☐	☐	☐	☐	☐

Other (please specify)

Please specify the specially trained professionals, if applicable (e.g. genetic nurse, midwives, physician assistants)

[ ]

* 6. WHICH HEALTH CARE PROFESSIONALS AT YOUR INSTITUTION ARE INVOLVED IN THE POST-TEST MANAGEMENT OF INDIVIDUALS HAVING A POSITIVE GENETIC TEST RESULTS? (Select one or more answers for each genetic test)

	Professionals who have performed risk assessment	Professionals who have prescribed genetic counselling	Professionals who have prescribed genetic testing	Professionals who are involved in treatment and surveillance of the genetic disorder	Other
BRCA1/2	☐	☐	☐	☐	☐
LYNCH SYNDROME	☐	☐	☐	☐	☐
FAMILIAL THROMBOPHILIA	☐	☐	☐	☐	☐
FAMILIAL HYPERCHOLESTEROLEMIA	☐	☐	☐	☐	☐

Other (please specify)

[ ]

* 7. WHO IS RESPONSIBLE FOR GENETIC TESTING OFFERED TO RELATIVES OF PROBANDS (CASCADE TESTING) IN YOUR INSTITUTION? (Select one or more answers for each genetic test)

	General practitioner	Medical geneticist	Genetic counsellor	Oncologist	Gynecologist	Specially-trained professionals	Other
BRCA1/2	☐	☐	☐	☐	☐	☐	☐
LYNCH SYNDROME	☐	☐	☐	☐	☐	☐	☐
FAMILIAL THROMBOPHILIA	☐	☐	☐	☐	☐	☐	☐
FAMILIAL HYPERCHOLESTEROLEMIA	☐	☐	☐	☐	☐	☐	☐

Other (please specify)

[ ]

Please specify the specially trained professionals, if applicable (e.g. genetic nurse, midwives, physician assistants)

[ ]

* 8. HOW ARE RELATIVES OF PROBANDS CONTACTED FOR GENETIC RESULTS AND TESTING? (Select one or more answers for each genetic test)

	The genetic service asks the proband for permission to contact relatives directly	The genetic service asks the proband for permission to contact relatives via a physician	Genetic counsellor	Oncologist	Gynecologist	Specially-trained professionals	Other
BRCA1/2	☐	☐	☐	☐	☐	☐	☐
LYNCH SYNDROME	☐	☐	☐	☐	☐	☐	☐
FAMILIAL THROMBOPHILIA	☐	☐	☐	☐	☐	☐	☐
FAMILIAL HYPERCHOLESTEROLEMIA	☐	☐	☐	☐	☐	☐	☐

Other (please specify)

Please specify the specially trained professionals, if applicable (e.g. genetic nurse, midwives, physician assistants)

SAPIENZA UNIVERSITÀ DI ROMA
Dipartimento Sanità Pubblica e Malattie Infettive

McGill
CGP Centre of Genomics and Policy
Centre de génomique et politiques

IDENTIFICATION OF DELIVERY MODELS FOR THE PROVISION OF PREDICTIVE GENETIC TESTING IN THE PROVINCE OF QUEBEC

PART 1. GENETIC TESTING

C. GENETIC SERVICE DELIVERY MODELS

* 1. WHICH OF THE FOLLOWING HEALTH CARE PROFESSIONALS IN YOUR INSTITUTION HAS **THE MOST PROMINENT ROLE** IN GENETIC TEST PROVISION AND COORDINATES TREATMENT AND SURVEILLANCE OF PATIENTS IN A MULTIDISCIPLINARY TEAM? (Select one answer for each genetic test)

	Medical geneticist	Primary care physicians	Other medical specialists	Physicians engaged in population screening programs	Other
BRCA1/2	○	○	○	○	○
LYNCH SYNDROME	○	○	○	○	○
FAMILIAL THROMBOPHILIA	○	○	○	○	○
FAMILIAL HYPERCHOLESTEROLEMIA	○	○	○	○	○

Other (please specify)

[ ]

Other medical specialists: e.g. radiologists, cardiologists, neurologists, gastroenterologists, oncologists, etc.
Population screening programs: e.g. breast cancer screening, colorectal cancer screening, newborn screening, etc.

* 2. WHICH OF THE FOLLOWING PATIENT PATHWAYS ARE ASSOCIATED TO THE PROVISION OF GENETIC TESTING IN YOUR INSTITUTION? (Select one or more answers for each genetic test)

	Patient → General practitioner or Medical specialist → Counsellor → Lab	Patient → General practitioner or Medical specialist → Lab	Patient → Counsellor → Lab	Patient → Lab	Other
BRCA1/2	☐	☐	☐	☐	☐
LYNCH SYNDROME	☐	☐	☐	☐	☐
FAMILIAL THROMBOPHILIA	☐	☐	☐	☐	☐
FAMILIAL HYPERCHOLESTEROLEMIA	☐	☐	☐	☐	☐

Other (please specify)

* 3. DOES YOUR INSTITUTION PROVIDE GENETIC COUNSELLING AND FOLLOW-UP SERVICES TO PATIENTS INTERESTED IN ORDERING GENETIC TESTS OVER THE INTERNET?

- ◯ Yes
- ◯ No

* 4. HAS THE IMPLEMENTATION OF GENETIC SERVICES FOR PREDICTIVE GENETIC TESTING DETERMINED THE DEVELOPMENT OF NEW PROFESSIONAL QUALIFICATIONS (e.g. genetic nurse with specific education and training in genetics)?

- ◯ Yes
- ◯ No

IF YES, PLEASE SPECIFY THE NEW PROFESSIONAL QUALIFICATIONS

BRCA1/2

LYNCH SYNDROME

FAMILIAL THROMBOPHILIA

FAMILIAL HYPERCHOLESTEROLEMIA

* 5. PLEASE LIST AT LEAST THREE FACTORS FOR EACH GENETIC TEST THAT FACILITATED THE IMPLEMENTATION OF THE CURRENT GENETIC SERVICE DELIVERY MODEL(S) IN YOUR INSTITUTION

BRCA1/2

LYNCH SYNDROME

FAMILIAL THROMBOPHILIA

FAMILIAL HYPERCHOLESTEROLEMIA

* 6. PLEASE LIST AT LEAST THREE CRITICAL ISSUES FOR EACH GENETIC TEST THAT ARE ALSO IMPLEMENTATION BARRIERS OF THE CURRENT GENETIC SERVICE DELIVERY MODEL(S) IN YOUR INSTITUTION

BRCA1/2

LYNCH SYNDROME

FAMILIAL THROMBOPHILIA

FAMILIAL HYPERCHOLESTEROLEMIA

7. PLEASE MAKE PROPOSALS FOR THE IMPROVEMENT OF THE EXISTING GENETIC SERVICE DELIVERY MODEL(S) FOR THE PROVISION OF PREDICTIVE GENETIC TESTING IN YOUR INSTITUTION OR PROVIDE ANY OTHER COMMENTS YOU WISH TO MAKE

SAPIENZA
UNIVERSITÀ DI ROMA
Dipartimento Sanità Pubblica e Malattie Infettive

McGill
CGP Centre of Genomics and Policy
Centre de génomique et politiques

IDENTIFICATION OF DELIVERY MODELS FOR THE PROVISION OF PREDICTIVE GENETIC TESTING IN THE PROVINCE OF QUEBEC

PART 2. ASSESSMENT OF GENETIC SERVICE DELIVERY MODELS

In this section you are kindly asked to answer questions on assessment of genetic service delivery models. The aim is to describe the flow and management of health information from each health facility to the provincial agencies where aggregate data are produced for the province of Quebec and used for planning activities.

A. EVALUATION OF ACTIVITY

* 1. DO YOU DEAL WITH OR HAVE GOOD KNOWLEDGE OF HEALTH DATA COLLECTION AND ANALYSIS AT YOUR INSTITUTION?

- ◯ Yes
- ◯ No

2. IF YES, IN WHAT TYPE OF INSTITUTION DO YOU WORK?

- ◯ Health facility
- ◯ Provincial agency
- ◯ Do not wish to specify
- ◯ Other (please specify)

* 3. DOES YOUR INSTITUTION USUALLY COLLECT, STORE AND RETRIEVE DATA ON GENETIC SERVICES?

- ◯ Yes
- ◯ No
- ◯ I am not aware/not certain

4. IF YES, IS THE PROCESS OF COLLECTION, STORAGE AND RETRIEVAL OF DATA ELECTRONIC?

- ◯ Yes
- ◯ No
- ◯ Under development
- ◯ I am not aware/not certain

* 5. IS THERE AN INFORMATION FLOW DIRECTING DATA FROM GENETIC SERVICES IN YOUR INSTITUTION TO PROVINCIAL OR NATIONAL LEVEL IN ORDER TO SUPPORT ACTIVITIES SUCH AS HEALTH PLANNING, CONTROL OR EVALUATION PROCESS?

- ◯ Yes
- ◯ No
- ◯ Yes, only to provincial level
- ◯ Yes, only to national level
- ◯ I am not aware/not certain

* 6. IS IT POSSIBLE TO KNOW HOW MANY GENETIC TESTS ARE PERFORMED PER YEAR IN QUEBEC, AT GENETIC SERVICE AND PROVINCIAL LEVELS?

- ◯ Yes
- ◯ No
- ◯ Yes, only at genetic service level
- ◯ Yes, only at provincial level
- ◯ I am not aware/not certain

* 7. IS INFORMATION ABOUT THE NUMBER OF GENETIC TESTS PERFORMED IN ASSOCIATION WITH GENETIC COUNSELLING PER YEAR AVAILABLE AT GENETIC SERVICES AND PROVINCIAL LEVELS?

- ◯ Yes
- ◯ No
- ◯ Yes, only at genetic service level
- ◯ Yes, only at provincial level
- ◯ I am not aware/not certain

* 8. WHAT OTHER MEASURES OF ACTIVITY OF GENETIC SERVICES ARE AVAILABLE IN QUEBEC? (Select one or more answers)

- [ ] Number of families/individuals seen
- [ ] Districts of residence of patient and family members
- [ ] Number of patients seen at central /peripheral clinics
- [ ] Number of new and follow-up appointments
- [ ] Number and type of individual genetic diagnoses
- [ ] Sources of referral (general practitioners, pediatricians, obstetricians, nurses, self referrals, other)
- [ ] Other (please specify)

IDENTIFICATION OF DELIVERY MODELS FOR THE PROVISION OF PREDICTIVE GENETIC TESTING IN THE PROVINCE OF QUEBEC

PART 2. ASSESSMENT OF GENETIC SERVICE DELIVERY MODELS

B. QUALITY ASSESSMENT

* 1. WHICH OF THE FOLLOWING MEASURES ARE USED TO ASSESS THE QUALITY OF GENETIC SERVICES IN QUEBEC? (Select one or more answers)

- ☐ Use of protocols of care
- ☐ Accuracy of diagnosis
- ☐ Accuracy of pedigree analysis
- ☐ Accuracy of risk assessment
- ☐ Quality of record keeping
- ☐ Quality and promptness of explanatory letters to referring clinicians and patients
- ☐ Agreed plans for follow-up of patients
- ☐ Arranging of prenatal tests and post termination counselling
- ☐ Identification of laboratories and units with which the clinical genetic unit can connect
- ☐ Participation of laboratories in accepted quality assurance schemes
- ☐ Patient satisfaction
- ☐ Other (please specify)

* 2. ARE PATIENTS INVOLVED IN THE ASSESSMENT OF GENETIC SERVICES IN QUEBEC THROUGH THE USE OF PATIENT-REPORTED OUTCOMES?

- ○ Yes
- ○ No
- ○ I am not aware/not certain

3. IF YES, WHICH AREAS RELATED TO PATIENTS ARE INVESTIGATED IN THE EVALUATION OF GENETIC SERVICES IN QUEBEC? (Select one or more answers)

- [ ] Knowledge (e.g. patient's knowledge about genetic risk, disease and genetic testing process and implications)
- [ ] Perception of risk (e.g. patient's perception of disease risk and of benefits and risk of genetic testing)
- [ ] Decision making (e.g. patient's intention to act on shared program)
- [ ] Coping (e.g. patient's psychological adaptation to genetic information)
- [ ] Satisfaction (e.g. patient's satisfaction with genetic service staff, facilities, counselling, etc.)
- [ ] Quality of life (e.g. patient's physical and mental quality of life)
- [ ] Other (please specify)

IF NO, WHICH AREAS SHOULD BE INVESTIGATED IN QUEBEC IN YOUR OPINION?

C. EVALUATION OF HEALTH OUTCOMES

* 1. GENETIC SERVICES MAY CONTRIBUTE TO A REDUCTION IN THE MORBIDITY AND MORTALITY FROM THE COMPLICATIONS OF SOME GENETIC DISEASES. IS IT POSSIBLE TO ROUTINELY HAVE DATA ABOUT MORBIDITY AND MORTALITY RATE DUE TO SPECIFIC GENETIC DISORDERS IN QUEBEC?

- ○ Yes
- ○ No
- ○ I am not aware/not certain

* 2. IS IT POSSIBLE TO LINK DATA ABOUT MORBIDITY AND MORTALITY TO GENETIC SERVICE'S ACTIVITY AND USE THEM AS OUTCOME MEASURES IN QUEBEC?

- ○ Yes
- ○ No
- ○ Under development
- ○ I am not aware/not certain

IF YES, COULD YOU SPECIFY THE GENETIC DISEASE AND THE PROGRAM FOR WHICH IT IS POSSIBLE TO USE MORBIDITY AND MORTALITY AS OUTCOME MEASURES OF GENETIC SERVICES (e.g. mortality for colorectal cancer as outcome of a program aimed at identifying Lynch syndrome mutations carriers and include them in a prevention program)?

1. GENETIC DISEASE/PROGRAM - HEALTH STATUS OUTCOMES

2. GENETIC DISEASE/PROGRAM - HEALTH STATUS OUTCOMES

3. GENETIC DISEASE/PROGRAM - HEALTH STATUS OUTCOMES

D. ELECTRONIC RECORDS AND GENETIC INFORMATION

* 1. DO THE ELECTRONIC RECORDS CURRENTLY IMPLEMENTED IN QUEBEC (DSQ) INCLUDE GENETIC INFORMATION?

- Yes
- No
- Under development
- I am not aware/not certain

2. IF YES, IS IT POSSIBLE TO USE GENETIC AND NON-GENETIC INFORMATION STORED IN THE HEALTH RECORDS TO EVALUATE THE APPROPRIATENESS OF GENETIC SERVICE PROCEDURES TO PATIENT'S CONDITION OR DIAGNOSIS (e.g. using the health record to reconstruct the clinical pathway of a patient related to a particular disease in order to compare it to the recommended clinical pathway)?

- Yes
- No
- Under development
- I am not aware/not certain

* 3. ARE THERE OTHER POSSIBILITIES, DIFFERENT FROM HEALTH RECORDS, TO RECONSTRUCT A PATIENT'S PATHWAY TO EVALUATE ITS APPROPRIATENESS (such as manual link between databases of laboratories and other facilities through a patient code)?

- Yes
- No
- Under development
- I am not aware/not certain

If yes or under development, please specify

E. GENETIC SERVICES AND COVERAGE

* 1. WHAT ARE THE GENETIC CONDITIONS THAT RECEIVE GOOD COVERAGE BY GENETIC SERVICES IN QUEBEC?

1. GENETIC CONDITION

2. GENETIC CONDITION

3. GENETIC CONDITION

* 2. IN YOUR OPINION, WHAT IS THE **MAIN BARRIER** TO THE REALIZATION OF UNIVERSAL COVERAGE FOR GENETIC CONDITIONS IN QUEBEC?

- Guarantee equity in access to health services so that all those who need the services can get them
- Guarantee quality of health services so that they can be good enough to improve health
- Guarantee sufficient health care financial resources
- All of the above
- I am not aware/not certain
- Other (please specify)

SAPIENZA UNIVERSITÀ DI ROMA
Dipartimento Sanità Pubblica e Malattie Infettive

McGill
CGP Centre of Genomics and Policy
Centre de génomique et politiques

IDENTIFICATION OF DELIVERY MODELS FOR THE PROVISION OF PREDICTIVE GENETIC TESTING IN THE PROVINCE OF QUEBEC

PART 3. POLICY CONTEXT OF GENETIC TESTING AND RELATED SERVICES

In this section, you are kindly asked to answer questions on policies governing the use of genomics medicine, genetic testing and related services, and genetic education and training available for health care professionals in Quebec.

A. POLICY

* 1. ARE YOU ENGAGED IN POLICY PLANNING AND/OR RESEARCH ON GENETIC SERVICES?

◯ Yes

◯ No

2. IF YES, ARE YOU AWARE OF A PLAN OR STRATEGY AIMED AT PLANNING AND DESIGNING HEALTH AND SOCIAL SERVICES FOR RARE DISEASES IN QUEBEC?

◯ Yes

◯ No

◯ Under development

If yes, please specify

[ ]

* 3. ARE YOU AWARE OF SPECIFIC PROVINCIAL OR LOCAL GUIDELINES THAT CAN HELP HEALTH DEPARTMENTS ORGANIZE GENETIC SERVICES TO ACT NOT ONLY AS SERVICES, BUT ALSO AS RESEARCH AND EDUCATIONAL RESOURCES?

◯ Yes

◯ No

◯ Under development

If yes, please specify

[ ]

* 4. GIVEN THE CRITICAL IMPORTANCE OF QUALITY FOR GENETIC TESTING, IS ACCREDITATION AND PARTICIPATION OF GENETIC LABORATORIES IN EXTERNAL QUALITY ASSESSMENT (EQA) SCHEMES MANDATORY IN QUEBEC?

- ◯ Yes
- ◯ No

5. IF NO, IS ACCREDITATION AND PARTICIPATION OF THE LABORATORIES IN EQA SCHEMES PROMOTED?

- ◯ Yes
- ◯ No

If yes, please specify the promotional strategy

[ ]

* 6. ARE YOU AWARE OF LOCAL GUIDELINES OF LOCAL ETHICS COMMITTEES (ECS) ENGAGED IN THE EVALUATION OF RESEARCH PROTOCOLS INVOLVING BIOBANKS AND BIOLOGICAL MATERIALS?

- ◯ Yes
- ◯ No

If yes, please specify

[ ]

* 7. THE INTRODUCTION OF GENETIC SERVICES IN MAINSTREAM MEDICINE HAS DETERMINED THE DEVELOPMENT OF NON-MEDICAL HEALTHCARE PROFESSIONALS THAT PROVIDE SERVICES IN SUPPORT OF MEDICAL GENETICISTS (e.g. genetic counsellors, genetic nurses, technical staff trained in genetics). ARE YOU AWARE OF ANY LEGISLATION GOVERNING THE PRACTICE OF NON-MEDICAL HEALTHCARE PROFESSIONALS IN QUEBEC?

	Yes	No	Under development	Other
Genetic counsellors	◯	◯	◯	◯
Genetic nurses	◯	◯	◯	◯
Technical staff in genetic diagnostic laboratories	◯	◯	◯	◯

Other (please specify)

[ ]

* 8. ARE YOU AWARE OF A REGISTRATION OR ACCREDITATION SYSTEM FOR NON-MEDICAL STAFF TRAINED IN GENETICS?

- ◯ Yes
- ◯ No
- ◯ Under development

If yes, please specify the professional categories

* 9. ARE YOU AWARE OF ASSOCIATIONS OR SCIENTIFIC SOCIETIES FOR NON-MEDICAL STAFF TRAINED IN GENETICS?

- ◯ Yes
- ◯ No
- ◯ Under development

If yes, please specify the professional categories

IDENTIFICATION OF DELIVERY MODELS FOR THE PROVISION OF PREDICTIVE GENETIC TESTING IN THE PROVINCE OF QUEBEC

PART 3. POLICY CONTEXT OF GENETIC TESTING AND RELATED SERVICES

B. GENETIC SERVICES: ACCESS AND AVAILABILITY

* 1. ARE LABORATORIES FOR GENETIC TESTING MOSTLY IN THE PUBLIC HEALTH SECTOR?

- ◯ Yes
- ◯ No

* 2. ARE THERE GENETIC TESTS OF PROVEN EFFICACY AND RELATED SERVICES COVERED BY PUBLIC HEALTH INSURANCES PROVIDED TO PATIENTS?

- ◯ Yes
- ◯ No

If yes, please specify

____

* 3. LOCATION AND DISTANCE FROM GENETIC SERVICES CONTINUES TO POSE A CRITICAL ACCESS BARRIER FOR INDIVIDUALS WHO LIVE IN RURAL AREAS. HAVE NEW APPROACHES BEEN DEVELOPED TO MEET THE DEMAND FOR GENETIC SERVICES OF UNDER SERVED POPULATIONS (e.g. telemedicine)?

- ◯ Yes
- ◯ No
- ◯ Under development

If yes or under development, please specify

____

* 4. IN YOUR OPINION, DOES THE CURRENT PROVISION OF GENETIC SERVICES ADEQUATELY MEET THE POPULATION NEEDS IN QUEBEC, IN TERMS OF ACCESS AND AVAILABILITY?

- ○ Yes
- ○ No

IF YES, PLEASE SPECIFY THE NEEDS YOU BELIEVE ARE CURRENTLY BEING MET

IF NO, PLEASE SPECIFY THE NEEDS YOU BELIEVE ARE CURRENTLY NOT BEING MET

* 5. WHAT ARE THE BIGGEST ISSUES HEALTH CARE PROVIDERS (physicians, nurses, laboratory staff, genetic counsellors, etc.) ARE FACING WITH RESPECT TO THE PROVISION OF GENETIC SERVICES IN QUEBEC? (Select one or more answers)

- ☐ Lack of integration between genetics and overall health care system
- ☐ Lack of education about genetics for health care providers
- ☐ Low public genetic literacy
- ☐ Lack of adequate genetic facilities (e.g. testing laboratories)
- ☐ Lack of research funding and reimbursement
- ☐ Other (please specify)

* 6. WHAT COULD PUBLIC HEALTH PROFESSIONALS DO TO BETTER SUPPORT HEALTH CARE PROVIDERS IN THE PROVISION OF GENETIC SERVICES IN QUEBEC? (Select one or more answers)

- ☐ Inform the general population about genetic services
- ☐ Inform policy makers about emerging genetic science and services
- ☐ Provide training and continuing education for healthcare professionals
- ☐ Develop standards of care (e.g. evidence-based guidelines)
- ☐ Provide policy guidance concerning Ethical, Legal, and Social Issues (ELSI)
- ☐ Other (please specify)

* 7. ARE YOU AWARE OF A LEGISLATION THAT SPECIFICALLY ADDRESSES DIRECT TO CONSUMER (DTC) GENETIC TESTING?

- ○ Yes
- ○ No
- ○ If yes, please specify

IF YES, IS DIRECT TO CONSUMER (DTC) GENETIC TESTING STRICTLY BANNED BY THE LEGISLATION(S)?

- ○ Yes
- ○ No

8. PLEASE PROVIDE ANY OTHER COMMENTS YOU WISH TO MAKE REGARDING THE POLICY OF GENETIC TESTING AND RELATED SERVICES AND/OR THE ROLE OF GENETICS IN THE PUBLIC HEALTH SYSTEM IN QUEBEC

C. PROFESSIONAL EDUCATION AND TRAINING

* 1. IMPROVING THE TRAINING OF HEALTH CARE PROFESSIONALS WILL ENABLE THEM TO RECOMMEND THE USE OF GENOMIC-BASED APPLICATIONS AND GENETIC SERVICES TO THEIR PATIENTS. IS PROFESSIONAL EDUCATION IN PUBLIC HEALTH GENOMICS AND ITS ETHICAL, LEGAL, AND SOCIAL IMPLICATIONS PROVIDED?

- ○ Yes
- ○ No

* 2. WHICH PROFESSIONAL CATEGORIES ARE THE COURSES IN PUBLIC HEALTH GENOMICS ADDRESSING? (Select one or more answers)

- ☐ Physicians
- ☐ Nurses
- ☐ Lab technicians
- ☐ Genetic counsellors
- ☐ Other (please specify)
